# Boldenone Undecylenate-Mediated Hepatorenal Impairment by Oxidative Damage and Dysregulation of Heat Shock Protein 90 and Androgen Receptors Expressions: Vitamin C Preventive Role

**DOI:** 10.3389/fphar.2021.651497

**Published:** 2021-04-27

**Authors:** Amany Behairy, Wafaa A. M. Mohamed, Lamiaa L. M. Ebraheim, Mohamed Mohamed Soliman, Yasmina M. Abd-Elhakim, Nabela I. El-Sharkawy, Taghred M. Saber, Maha M. El Deib

**Affiliations:** ^1^ Department of Physiology, Faculty of Veterinary Medicine, Zagazig University, Zagazig, Egypt; ^2^ Department of Clinical Pathology, Faculty of Veterinary Medicine, Zagazig University, Zagazig, Egypt; ^3^ Department of Histology and Cytology, Faculty of Veterinary Medicine, Zagazig University, Zagazig, Egypt; ^4^ Clinical Laboratory Sciences Department, Turabah University College, Taif University, Taif, Saudi Arabia; ^5^ Department of Forensic Medicine and Toxicology, Faculty of Veterinary Medicine, Zagazig University, Zagazig, Egypt; ^6^ Department of Biochemistry, Faculty of Veterinary Medicine, Zagazig University, Zagazig, Egypt

**Keywords:** boldenone undecylenate, vitamin C, hepatorenal damage, oxidative stress, heat shock protein 90, androgen receptors

## Abstract

Boldenone Undecylenate (BLD) is a synthetic derivative of testosterone and a widely used anabolic androgenic steroid. The health risk of BLD use as a pharmaceutical or dietary supplement is still underestimated and under-reported. Vitamin C (VC) has been recognized as an antioxidant with prominent hepatorenal protective effects. This study investigated the possible preventive activity of VC against BLD-induced hepatorenal damage. Forty adult male Wistar rats were classified into five groups: control, vehicle control, VC (orally given 120 mg/kg b. wt./day), BLD (intramuscularly injected 5 mg/kg b. wt./week), and BLD + VC-treated groups. The experiment continued for eight weeks. Serum levels of alanine aminotransferase (ALT) and aspartate aminotransferase (AST) were measured. Serum contents of total protein (TP), albumin (ALB), globulin, total cholesterol (TC), triglycerides (TG), high-density lipoprotein-cholesterol (HDL-C), low-density lipoprotein-cholesterol (LDL-C), and very-low-density lipoprotein–cholesterol (VLDL-C) were also assayed. Urea, creatinine, and uric acid levels were determined together with sodium and potassium electrolytes measuring. Moreover, oxidative stress indicators including reduced glutathione (GSH), glutathione peroxidase (GPx), glutathione-S-transferase (GST), and glutathione reductase (GSR) as well as malondialdehyde (MDA) levels were measured in both hepatic and renal tissues. Corresponding histological examination of renal and hepatic tissues was conducted. Besides, immunohistochemical evaluations for androgen receptors protein (AR) and heat shock protein 90 (Hsp 90) expressions were performed. BLD caused significant rises in serum ALT, AST, TP, ALB, TC, TG, LDL-C, VLDL-C, urea, creatinine, uric acid, potassium, and MDA levels. Further, BLD-injected rats showed significant declines in the serum levels of HDL-C, sodium, GSH, GPx, GST, and GSR. Besides, distinct histopathological perturbations were detected in renal and hepatic tissues of BLD-injected rats. AR and Hsp 90 immunoexpression were increased in hepatic and renal tissues. In contrast, VC significantly reversed the BLD-induced hepatorenal damage in co-treated rats but not ameliorated AR protein overexpression. VC could be an efficient preventive supplement for mitigating BLD-induced hepatorenal damage, possibly via controlling oxidative stress events.

## Introduction

The use of anabolic–androgen steroids (AASs) has recently increased among amateur and men who are not athletes but want to improve their physical appearance. Unfortunately, AASs use is associated with many adverse effects, of which some are serious and hard to cope with (Gagliano-Jucá and Basaria, 2019). Boldenone undecylenate or boldenone undecenoate (BLD), a well-known AASs member, is primarily produced for veterinary use mainly for horses and known as Equipoise, Ganabol, Equigan, and Ultragan (Tousson et al., 2016). It also increases the growth and nutrient conversion of food-producing animals. Despite being banned in humans, BLD is still available illegally and heavily used by athletes and bodybuilders and for fitness purposes in non-athletics (Park et al., 2019). Also, the illegal use of BLD in racing horses and food-producing animals still represents a major concern (Le Bizec et al., 2006; Genangeli et al., 2019). Several earlier reports confirmed the detection of BLD and its metabolites in humans and calves urine samples (Buiarelli et al., 2005; Le Bizec et al., 2006; Wu et al., 2015). Consequently, BLD could adversely affect human directly by injecting muscles and indirectly by eating meat from BLD-treated animals (Oda and El-Ashmawy, 2012).

Testosterone and AASs pass across the target cell membrane into the blood system and are connected to intra-cytoplasmic receptors. This complex is then moved to the cell nucleus, where it binds with DNA. It produces RNA and subsequently increases protein synthesis in muscles (Barceloux and Palmer, 2013). At the same time, AASs alter the anabolic/androgenic function, androgen receptors (AR) binding affinity, and metabolism (Pope et al., 2014). The 17-beta-hydroxy group of injectable AASs, like BLD, is esterified, yielding more lipid-soluble products slowly released into the blood (Liddle and Connor, 2013). Several health disorders have been associated with BLD misuse like renal damage (Barakat et al., 2015), cardiovascular disorders (Tousson et al., 2016), liver dysfunction (Ziaolhagh et al., 2018), and testicular problems (Behairy et al., 2020b).

Since the liver is the crucial organ in the metabolism of drugs and the kidneys account for their excretion, the high doses of AASs usually impact these organs (Frankenfeld et al., 2014). Hepatotoxicity of AASs is linked to increased infiltration in hepatic tissue by neutrophils, lymphocytes, and eosinophils (Neri et al., 2011; Bond et al., 2016). Several liver alterations have been reported following AASs abuse, like subcellular hepatocyte modifications, hepatocellular hyperplasia, and general liver damage (Solimini et al., 2017). Also, chronic kidney illness remains long asymptomatic before diagnosis in AASs users. Besides, because of its growing muscle mass, creatinine levels are often high in AASs-treated patients, even in the absence of renal injury (Parente Filho et al., 2020). The increase in urea concentration with the AASs use is attributed to the severe reduction in kidney function (Herlitz et al., 2010). Nephrosclerosis, disruptive glomerulosclerosis, and acute renal failure have been observed in AASs users (Taher et al., 2008; Winnett et al., 2011). BLD has been reported to enhance the retention of nitrogen, protein synthesis, appetite, and erythropoietin release in the kidneys but decreases protein degradation. Besides, nitrogen, body water, sodium (Na^+^), potassium (K^+^), and calcium ions are stored (Gabr et al., 2009).

Oxidative stress has been strongly implicated in BLD-induced hepatic and nephrotoxicity. In this regard, El-Moghazy et al. (2012) and Farag et al. (2015) reported that BLD injection resulted in liver and kidney oxidative stress as revealed by disturbed superoxide dismutase (SOD), glutathione, and malondialdehyde (MDA) levels. Heat shock proteins (HSPs) are a critical part of the cell stress response to injury reduction, rapid recovery, and homeostasis (Atalay et al., 2009). Hsp90 is one of the most commonly recognized Hsps that refers to a chaperone protein, which allows other molecules to fold correctly and stabilize proteins against heat stress. It also controls the cell proteins growth (Tukaj and Węgrzyn, 2016) and facilitates intracellular transport, protein degradation, and cell signaling (Pearl, 2016). Moreover, Hsp90 play a vital role in protecting cells from various stress conditions (Parcellier et al., 2003). In particular, several earlier reports confirmed the strong link between Hsp90 over-expression and oxidative stress conditions (Profumo et al., 2018; Castro et al., 2019). In this regard, AASs induced oxidative-stress has been earlier reported to trigger Hsp90 upregulation in the kidney tissues of nandrolone decanoate (ND)-treated mice (Riezzo et al., 2014).

In these years, great concern has been paid to using natural antioxidants as a prophylactic or therapeutic agent against side effects of medication misuse (Mohamed et al., 2019; El-Rahman et al., 2020; Abd-Elhakim et al., 2021). Vitamin C (VC) is an essential micronutrient and important nutritional supplement (Bozonet et al., 2015). Vitamin C tablets are a commonly used and widely available oral supplement that highly consumed throughout the world (Granger and Eck, 2018; Cerullo et al., 2020). VC is an outstanding electron source that donates electrons to free radicals such as superoxide and hydroxyl radicals and quenches their responsiveness both inside and out of cells (Bindhumol et al., 2003). VC rescued insecticide-induced hepatic toxicity (Abd-El-Ghaney, 2002). VC also rescued nonsteroidal anti-inflammatory drug-induced acute kidney injury in rats by enhancing kidney function and reducing renal tissue inflammation and oxidative damage (El-Shafei and Saleh, 2016).

Based on VC's antioxidant activities, this study explored the ability of VC oral dosing to mitigate hepatorenal damage caused by BLD. Different biochemical markers have been measured for liver and kidney function assessment. Besides, histopathological and histochemical examinations of the hepatic and renal AR and Hsp90 immunoexpression were performed to understand BLD impairment mechanisms and possible VC protective role.

## Material and Methods

### Chemicals

A commercial form of BLD (1,4-androstadiene-17b-ol-3-one) named Equigan® (Lab Tornel, Co., Mexico) was used. Each 1 ml contains 50 mg BLD in 1 ml sesame oil. VC was purchased as tablets (500 mg ascorbic acid/tablet) (Kahira Pharma Co., Cairo, Egypt). VC was freshly prepared directly before daily dosing, by dissolving the tablets in distilled water to the required concentration, to overcome the problem of the instability and mimic practical application in humans. All the other chemicals and reagents used were of analytical quality and bought by Sigma-Aldrich Co.

### Animals Housing and Caring

Forty adult male Wistar rats (10–12 weeks age; 160 ± 10 g) were obtained from the Laboratory Animal Research Unit, Faculty of Veterinary Medicine, Zagazig University. Rats were familiarized with experimental conditions for two weeks earlier treatment. The rats were kept in metal cages and were given a basal diet and water ad libitum. All rats have been maintained in standard environmental conditions (12 h light/dark cycles, 40–60% relative humidity, and 23°C room temperature). Following the National Institutes of Health's guiding principles for treating research animals, the institute for animal ethics approved all protocols.

### Experimental Protocol

The rats were randomly divided into five experimental groups of eight rats each. The control group: in which rats were administered distilled water orally. The vehicle control group: rats were intramuscularly injected with 0.25 ml sesame oil/kg b. wt. once a week. The VC treated group: in which rats were orally gavaged with 120 mg VC/kg b. wt. daily. The BLD treated group: rats were intramuscularly injected with 5 mg BLD/kg b. wt. once a week (Bueno et al., 2017). The BLD + VC co-treated group was given BLD and VC at the same mentioned dose and route. The trial continued for eight weeks.

### VC Dose Selection

The tested dose of VC (120 mg VC/kg b. wt.) was chosen based on the dose that was seen to be most effective in lowering toxicity induced by various xenobiotic and oxidative stress associated conditions in several earlier studies (Shahidi et al., 2008; Moosavirad et al., 2016; Abu Zeid et al., 2018). In addition, several earlier animal studies have tested VC beneficial therapeutic roles at low doses (10–50 mg/kg bwt) and at higher doses (100–400 mg/kg bwt)(Shahidi et al., 2008; Shivavedi et al., 2019). In these studies, the higher dose VC showed better effects in organ protection and in the improvement of survival. Additionally, based on the tolerable upper intake levels for VC in human (up to 2000 mg) (Institute of Medicine. Food and Nutrition Board, 2000), an animal equivalent dose up to 200 mg/kg can be used when converted following the guidance of the United States Food and Drug Administration (United States Food Drug Administration, 2005; Nair and Jacob, 2016).

### Sampling

At the end of eight weeks, rats in the different experimental groups were fasted overnight, weighted, and blood samples were collected from the retro-orbital plexus into test tubes without anticoagulant. The collected samples were left for 30 min to clot at room temperature; then, the serum was separated by centrifugation (3000 rpm, 20 min). The serum was stored at −20°C until analyzed for liver and kidney function tests. Rats were euthanized by decapitation. Then, the liver and kidney were necropsied and cleaned with normal saline. Specimens from each organ were separated into three parts. Two parts were weighted and homogenized separately (10% w/v) with a “Potter-Elvehjem”-type glass homogenizer (Thomas Scientific, NJ, United States). One part was homogenized in phosphate buffer saline (PBS) 50 mM pH (7.4) for estimation of protein content, GST and GSR enzymes activities, and GSH level. The second part was homogenized in potassium phosphate buffer 10 mM pH (7.4) for estimation of MDA level. The crude tissue homogenate was centrifuged at 5000 × g for 15 min in cold centrifuge (centurion scientific Ltd., K2015R, United Kingdom). The resultant supernatant was filtered using 0.45 μm millipore filter to remove any tissue debris then preserved at −20°C to evaluate oxidative stress and lipid peroxidation markers. Protein content in tissue homogenate was measured according to the Lowry method (Waterborg, 2009). Then the third parts were kept in 10% buffered neutral formalin for further histopathological and immunohistochemical evaluations.

### Serum Biochemical Analysis

ALT and AST activities were assessed by Bergmeyer et al. (1978) method. Total protein (TP) and albumin (ALB) amounts were estimated by Diamond Diagnostics kits (Cairo, Egypt) following the procedures of Henry (1964) and Doumas et al. (1971), respectively. The content of globulins has been determined by eliminating albumin from the total protein. Total cholesterol (TC), Total triglycerides (TG), and HDL-cholesterol (HDL-C) concentrations were estimated using reagent kits purchased from Spinreact Company (Spain) following the protocols of Deeg and Ziegenhorn (1983), Fossati et al. (1983), and Burstein et al. (1970), respectively. Very low-density lipoprotein cholesterol (VLDL-C) and low-density lipoprotein cholesterol (LDL-C) were calculated according to the formula of Friedewald et al. (1972). According to Kaplan (1984) and Fossati et al. (1983), urea and creatinine levels were assessed using commercial kits from BioMed Diagnostic Co., Cairo, Egypt. The uric acid level was evaluated in line with Barham and Trinder (1972) protocol. The electrolytes levels comprising Na^+^ and K^+^ were determined via Easylyte plus Na/K/Cl Analyzer (Medica Corporation, Netherland).

### Evaluation of Hepatic and Renal Oxidative Stress Markers

All oxidative stress variables were measured spectrophotometrically using Biodiagnostic kits (Cairo, Egypt). The reduced glutathione (GSH), glutathione peroxidase (Gpx), glutathione-S-transferase (GST) levels were determined according to the methods of Beutler et al. (1963), Paglia and Valentine (1967), and Habig et al. (1974), respectively. Glutathione reductase (GSR) was assayed according to the method of Goldberg (1984). MDA content was determined consistent with Ohkawa et al. (1979) method.

### Histopathological Examination

The formalin-fixed representative livers and kidneys specimens were dehydrated in ascending ethanol grades, cleared in xylene, and impregnated and embedded in paraffin. Five-microns thick tissue sections were prepared and stained with hematoxylin and eosin stains followed the protocol of Bancroft et al. (2018). The slides were then examined microscopically and the encountered histopathological changes were recorded. A quantitative lesion scoring in the hepatic and renal tissues was done according to the method described by Galal et al. (2019), and the results were expressed as percentages (means ± SD).

### Immunohistochemical Investigation

The immunohistochemical study involved staining of the AR and Hsp90 antigens in the hepatic and renal tissues by rabbit monoclonal anti-androgen receptor antibody [ER179 (2)] - ChIP Grade (ab108341) and mouse monoclonal anti-Hsp90 antibody [AC88] (ab13492) primary antibodies (Abcam, United Kingdom), and 3,30-Diaminobenzidine chromogen (DAB) according to the avidin-biotin-peroxidase complex protocol mentioned by Hsu et al. (1981). Besides, negative control sections were prepared using phosphate buffer saline as a substitute for the primary antibodies to demonstrate whether the IHC test is specific and avoid non-specific reactions and false-positive results (Hewitt et al., 2014; Torlakovic et al., 2015). DAB density is not proportional to epitope concentration, and most cells either in the livers or kidneys gave varying degrees of immunopositivity with both biomarkers. Therefore, the quantitative assessment of the AR and Hsp90 immunoexpressions was done by calculating DAB brown areas' fractions to the total areas of the images. Using the open-source ImageJ software version 1.41, five randomly selected, fixed-size microscopic images/organ/animal snapshotted at the same magnification (× 40), and the exposure time were captured. The results were denoted as means ± SD.

### Statistical Analysis

The data were presented as the mean ± SD. The difference between groups was statistically evaluated by One-way variance analysis (ANOVA), then a post-hoc test of Duncan was used for comparisons. Differences at *p* ≤ 0.05 were considered significant. Data obtained were plotted using Graphpad computer program (ISI Software, Philadelphia, PA) to perform regression analysis. The Shapiro-Wilk test was used to check all data for normality.

## Results

### Effects on Liver Function Parameters

As showed in Table 1, there was a significant increment in serum levels of ALT (191.97%) and AST (99.97%) enzymes in BLD-injected group relative to the control group. Nevertheless, the co-treatment with VC in BLD-injected rats significantly reduced the increment in ALT and AST enzyme levels comparable to the BLD-injected group. No significant change in serum ALT and AST were observed between the control and VC-treated group.

**TABLE 1:** Effect of boldenone (BLD) (5 mg/kg bwt/once a week) and/or vitamin C (VC) (120 mg/kg b.wt/daily) treatment for eight weeks on liver enzymes, lipid profile, kidney damage products, electrolytes, and protein profile of adult male Wistar rats.

	Control	Vehicle control	VC	BLD	BLD + VC
ALT (U/L)	12.33 ± 2.05	13.67 ± 1.70	12.67 ± 1.25	36.00^a^ ± 5.35	22.33^a^ ^,^ ^b^ ± 4.50
AST (U/L)	25.67 ± 2.05	21.67 ± 1.70	23.33 ± 3.86	51.33^a^ ± 3.86	37.00^a^ ^,^ ^b^ ± 2.94
TC (mg/dl)	138.67 ± 13.02	142.67 ± 8.34	146.00 ± 11.34	215.67^a^ ± 6.80	180.00^a^ ^,^ ^b^ ± 7.26
TG (mg/dl)	92.00 ± 3.27	87.33 ± 8.34	87.33 ± 2.87	133.33^a^ ± 6.34	108.67^a^ ^,^ ^b^ ± 10.66
HDL-C (mg/dl)	55.00 ± 5.72	57.33 ± 2.05	56.00 ± 5.10	32.33^a^ ± 2.05	44.33^a^ ^,^ ^b^ ± 2.49
LDL-C (mg/dl)	65.27 ± 10.00	67.87 ± 8.68	72.53 ± 11.20	156.67^a^ ± 8.98	113.93^a^ ^,^ ^b^ ± 6.70
VLDL-C (mg/dl)	18.40 ± 0.65	17.47 ± 1.67	17.47 ± 0.57	26.67^a^ ± 1.27	21.73^a^ ^,^ ^b^ ± 2.13
Urea (mg/dl)	22.33 ± 2.05	23.67 ± 2.49	23.00 ± 2.94	45.33^a^ ± 2.87	29.67^a^ ^,^ ^b^ ± 2.05
Creatinine (mg/dl)	0.69 ± 0.01	0.67 ± 0.01	0.68 ± 0.02	1.37^a^ ± 0.13	0.96^a^ ^,^ ^b^ ± 0.09
Uric acid (mg/dl)	5.50 ± 0.73	5.87 ± 0.78	5.53 ± 0.37	10.20^a^ ± 0.29	7.90^a^ ^,^ ^b^ ± 0.37
Na^+^ (mEq/L)	146.00 ± 3.74	152.00 ± 2.94	146.33 ± 3.40	103.00^a^ ± 6.68	115.67^a^ ^,^ ^b^ ± 13.20
K^+^ (mEq/L)	2.93 ± 0.25	3.13 ± 0.29	2.83 ± 0.34	7.40^a^ ± 0.49	4.90^a^ ^,^ ^b^ ± 0.78
TP (g/dl)	7.47 ± 0.45	7.60 ± 0.57	7.43 ± 0.74	8.73^a^ ± 0.12	7.80^b^ ± 0.08
ALB (g/dl)	4.17 ± 0.25	4.37 ± 0.24	4.20 ± 0.16	5.10^a^ ± 0.36	4.40^b^ ± 0.22
Globulin (g/dl)	3.30 ± 0.65	3.23 ± 0.53	3.23 ± 0.65	3.63 ± 0.41	3.40 ± 0.28
AG ratio	1.33 ± 0.38	1.38 ± 0.23	1.35 ± 0.28	1.43 ± 0.27	1.30 ± 0.18

ALB, albumin; ALT, alanine transaminase; AST, aspartate transaminase; AG ratio, albumin globulin ratio. HDL-C, high-density lipoprotein cholesterol; K^+^, potassium; LDL-C, low-density lipoprotein cholesterol; Na^+^, sodium; TC, total cholesterol; TG, triglycerides; TP, total protein; VLDL-C, very low-density lipoprotein cholesterol. A one-way analysis of variance (ANOVA) followed by Duncan's Multiple Range test was used for statistical analysis;

a Significantly different compared to the control group at *p ≤ 0.05*.

b Significantly different from the BLD treated group *p* ≤ 0.05. The values shown are means ± SD. *n* = 8.

Compared with the control group, a significant increase of TP (16.91%) and ALB (22.30%) was evidenced in BLD-injected group. The VC co-treatment with BLD revealed a significant reduction in TP and ALB compared with the BLD-injected group. No evidenced changes in TP and ALB values were observed between control and VC-treated rats. No significant differences were recorded in globulin contents and A/G ratio between all treated groups.

### Effects on Lipid Profiles

The BLD-injected group demonstrated significant increases in the TC, TG, LDL-C, and VLDL-C by 55.53%, 44.93%, 140.14%, and 44.95%, respectively, but a significant HDL-C reduction by 41.21% compared to control group (Table 1). The former increases were significantly minimized in the BLD + VC co-treated group compared with the BLD-injected group. Also, the co-treatment with VC significantly increased HDL-C in BLD-injected rats. No significant alterations were noticed in lipid profile indicators between the control and the VC-treated group.

### Effects on Renal Injury Indicators

The effects of intramuscular injection of BLD and oral VC administration for eight weeks on rats' serum renal injury markers are displayed in Table 1. A significant rise in serum levels of urea, creatinine, and uric acids by 103.01%, 98.19%, 85.45%, respectively, was observed in BLD-injected rats relative to the control ones. Nevertheless, the rats co-administered VC with BLD showed a significant reduction in the raised urea, creatinine, and uric acid levels compared to BLD-injected ones. No apparent changes in renal function variables were detected between control and VC-treated rats.

### Effects on Serum Electrolytes

As demonstrated in Table 1, the BLD injection for eight weeks in rats significantly increased K^+^ levels (152.56%) but reduced the Na^+^ levels (29.45%) compared with the control group. On the other hand, the VC co-treatment in BLD-injected rats significantly reduced the increase in K^+^ levels but restored Na^+^ concentrations compared to the BLD group. VC treatment exhibited no significant change in electrolytes levels compared to the control group.

### Effects on Hepatic and Renal Oxidative Stress Markers

**FIGURE 1:**
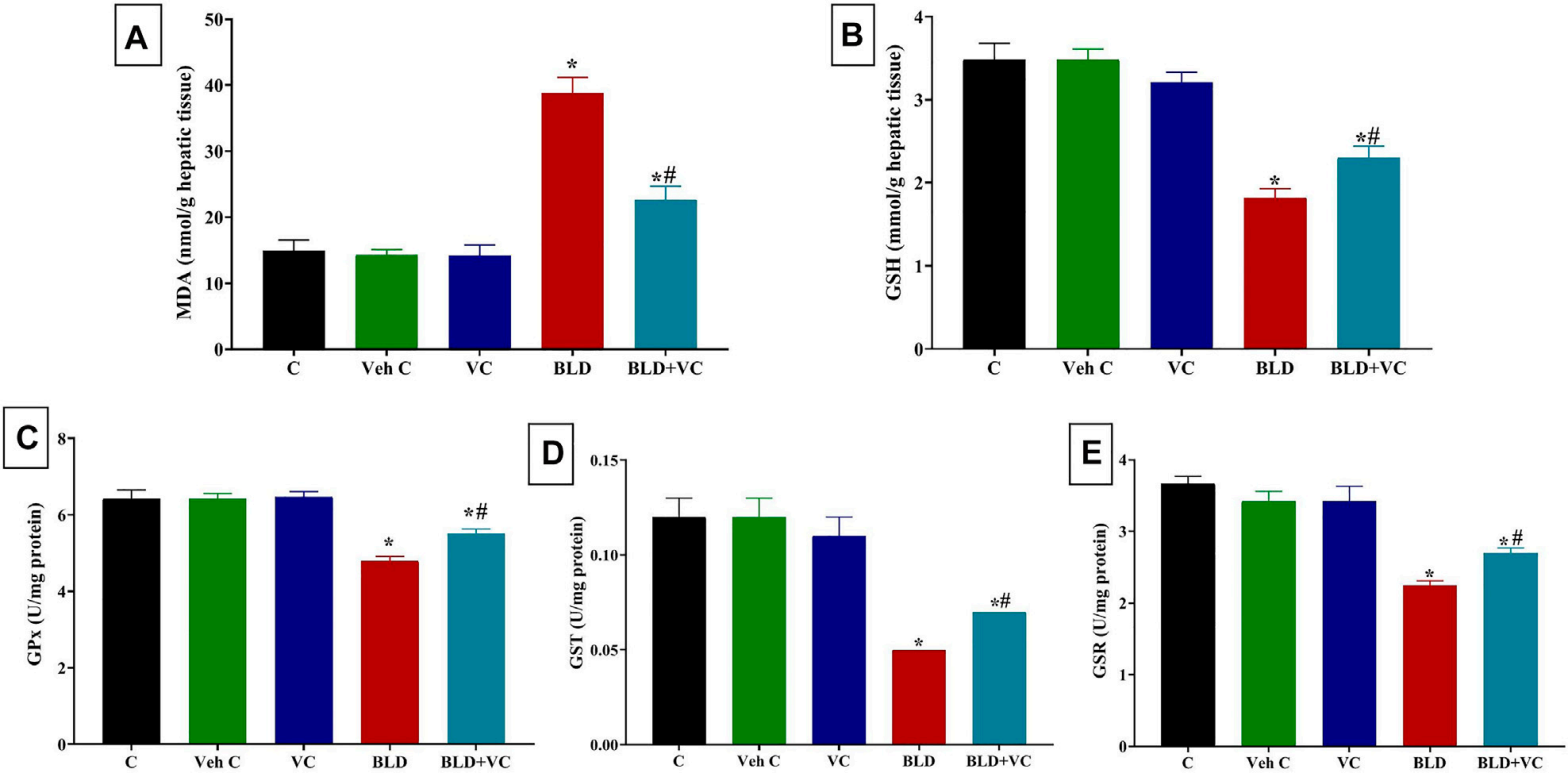
Changes in hepatic oxidative stress and lipid peroxidation indicators in boldenone (BLD) (5 mg/kg bwt/once a week, eight weeks) and/or vitamin C (VC) (120 mg/kg b. wt/daily, eight weeks) treated adult male Wistar rats. **(A)** Malondialdehyde, MDA; **(B)** Reduced glutathione, GSH; **(C)** Glutathione peroxidase, Gpx; **(D)** Glutathione-S-transferase, GST; **(E)** Glutathione reductase GSR. Data are expressed as the mean ± SD (*n* = 8 replicates). * Significantly different compared to the control group at *p ≤ 0.05*. ^#^ Significantly different from the BLD treated group at *p* ≤ 0.05.

**FIGURE 2:**
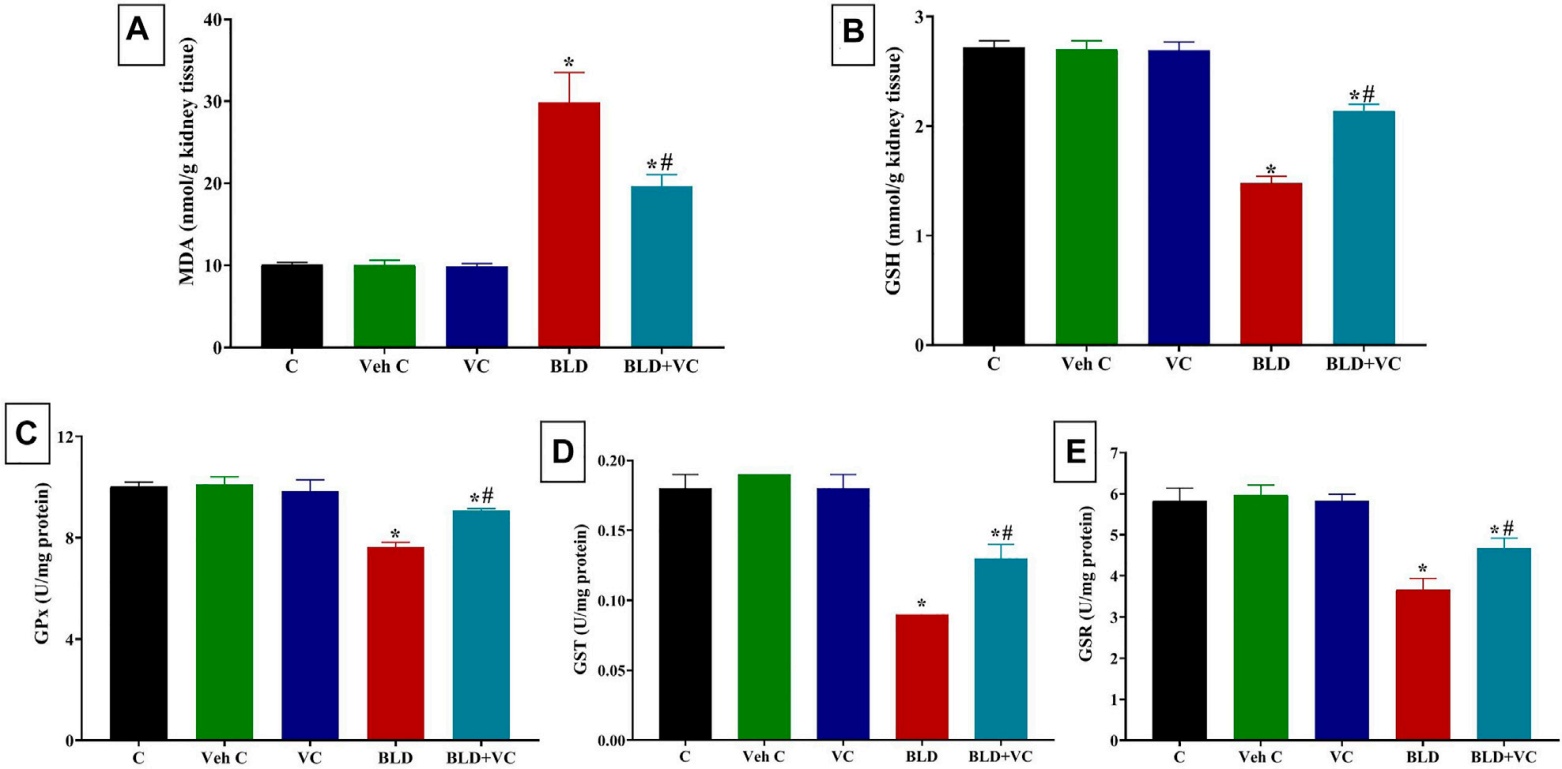
Changes in renal oxidative stress and lipid peroxidation indicators in boldenone (BLD) (5 mg/kg bwt/once a week, eight weeks) and/or vitamin C (VC) (120 mg/kg b. wt/daily, eight weeks) treated adult male Wistar rats. **(A)** Malondialdehyde, MDA; **(B)** Reduced glutathione, GSH; **(C)** Glutathione peroxidase, Gpx; **(D)** Glutathione-S-transferase, GST; **(E)** Glutathione reductase GSR. Data are expressed as the mean ± SD (*n* = 8 replicates). * Significantly different compared to the control group at *p* ≤ 0.05. ^#^ Significantly different from the BLD treated group at *p* ≤ 0.05.

As represented in Figures 1 and 2, rats orally administered VC for eight weeks revealed non-significant changes in hepatic and renal oxidant/antioxidant markers compared to the control group. Intramuscularly injected rats with BLD for eight weeks showed significantly higher MDA content (159.19% and 195.72%, respectively) but significantly lower content of GSH, GPx, GST, and GSR in the liver (47.70%, 25.35%, 58.33%, 38.76%, respectively) and kidney (45.68%, 23.90%, 50.97%, and 37.09%, respectively) tissues compared to control groups. However, the VC co-treatment in intramuscularly BLD injected rats significantly reduced the MDA level but increased GSH, GPx, GST, and GSR levels compared with BLD-injected group.

### Histopathological and Immunohistochemical Findings: Liver

**FIGURE 3:**
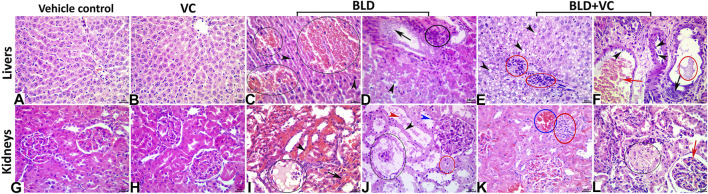
**(A–F)** Representative photomicrograph of the H&E stained hepatic tissue sections showing normal histological pictures in the vehicle control **(A)** and VC-treated **(B)** rats. The BLD-treated rats showing peliosis hepatis (black ellipses), and nuclear pyknosis (black arrowheads) **(C)**, biliary hyperplasia (black arrow), hydropic degeneration (black arrowhead), and portal inflammatory infiltrate (black ellipse) **(D)**. The BLD + VC-treated rats showing lipidosis (black arrowheads), portal inflammatory infiltrate (red ellipses) **(E)**, portal congestion (red arrow), endothelial hypertrophy (black arrowheads), biliary hyperplasia (black arrow), and cholestasis (red ellipse) **(F)**. The scale bar is 20 microns. **(G–L)** Representative photomicrograph of the H&E stained renal tissue sections showing normal histological pictures in the vehicle control **(G)** and VC-treated **(H)** rats. The BLD-treated rats showing glomerular necrosis with severe hypocellularity and an eosinophilic filtrate (black ellipse), interstitial congestion (black arrowhead), and hemorrhage (black arrow) **(I)**, glomerular necrosis (black ellipse), tubular attenuation (black arrowhead), pyknosis (blue arrowhead), necrosis (red ellipse), and luminal debris (red arrowhead) **(J)**. The BLD + VC-treated rats showing interstitial congestion (blue ellipse), and mononuclear cell aggregate (red ellipse) **(K)**, glomerular sclerosis (black ellipse), and congestion (red arrow), lipidosis (black arrowheads), portal inflammatory infiltrate (red ellipses) (E), portal congestion (red arrow) **(L)**. The scale bar is 20 microns.

**FIGURE 4:**
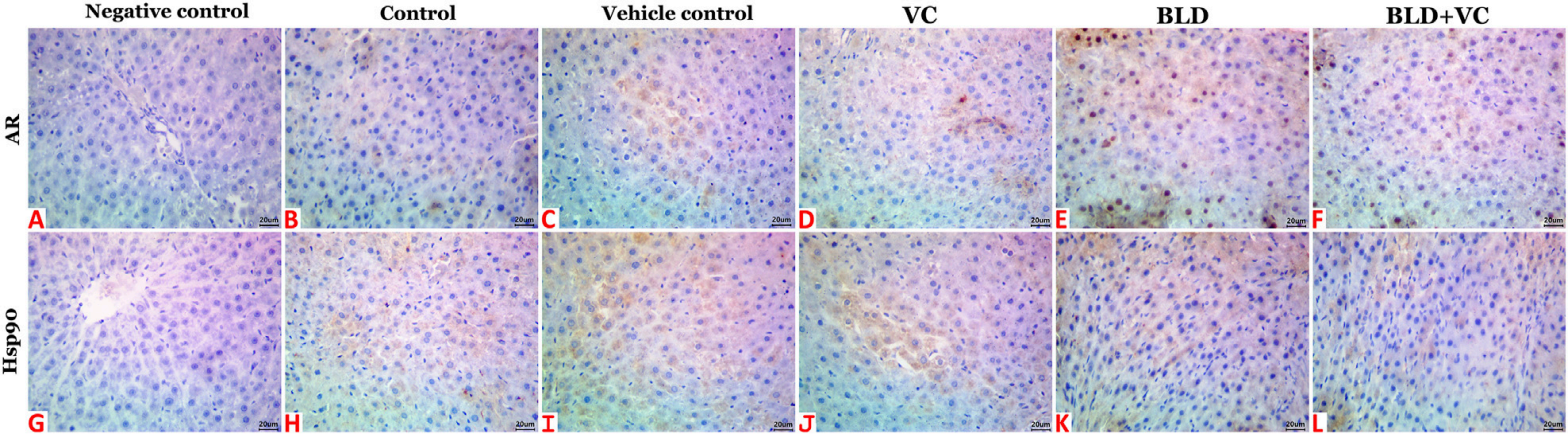
Representative photomicrograph of hepatic tissue sections of AR and Hsp90 immunoexpression showing a marked increase in the fractions of DAB brown areas in the BLD **(E and K)** and BLD + VC-treated **(F and L)** rats compared to the control **(B and H)**, sesame oil **(C and I)** and VC-treated **(D and J)** rats. **(A and G)** are negative controls. The scale bar is 20 microns.

Normal histological pictures were seen in the livers of the control, sesame oil, and VC-treated rats (Figures 3A,B). At the same time, those of the BLD-injected animals showed various structural alterations included cellular swelling, vacuolar and hydropic degenerations (centrally located nuclei with cytoplasmic vacuolations and ballooning), lipidosis (microvesicular and/or macrovesicular), and cavernous peliosis hepatis (several, randomly distributed, blood-filled spaces not lined by endothelium with direct contact between the blood and the neighboring hepatocytes which exhibited pyknotic changes) (Figure 3C). Parenchymal and/or mononuclear cell infiltrations were a common feature. Neither neoplastic nor preneoplastic changes were seen in the hepatocytes, but noticeable biliary hyperplasia (stratification of cholangiocytes with minimal nuclear atypia) was evident (Figure 3D). The hepatic tissue sections taken from BLD + VC co-treated animals manifested variable degrees of histological alteration. Generally, VC supplementation lowered the frequencies and extent of BLD-induced structural alterations, particularly peliosis hepatis and biliary hyperplasia but did not maintain the normal hepatic morphology. The most encountered hepatic lesions included lipidosis, vacuolations, portal congestions with endothelial hypertrophy and mononuclear cell infiltrations, and mild biliary hyperplastic changes, sometimes accompanied with cholestasis (Figures 3E,F). A quantitative lesion scoring in all groups was summarized in Table 2. Immunohistochemically, image analysis indicated that the AR and Hsp90 fractions of DAB brown areas in the hepatic tissue sections of the BLD-injected animals (AR, 7.76 ± 0.44; Hsp90, 10.91 ± 1.52) was significantly (*p* ≤ 0.0.5), increased compared to the control (AR, 5.66 ± 0.92; Hsp90, 7.52 c ± 0.46), sesame oil (AR, 5.31 ± 0.43; Hsp90, 7.54 ± 0.52), and VC (AR, 5.44 ± 0.47; Hsp90, 7.59 ± 0.41). Subjectively, BLD-injection increased the AR nuclear expression but decreased the cytoplasmic concentration of the receptor. In contrast, the increase in the Hsp90 expression was related to the increased cytoplasmic concentration with minor changes in the nuclear expression. VC supplementation significantly decreased the Hsp90 fractions of DAB brown areas. Yet, it had no significant effect on the AR fractions of DAB brown areas in the BLD + VC co-treated animals than the BLD-injected animals. A quantitative scoring of the AR and Hsp90 fractions of DAB brown areas in all groups was shown in (Figures 4A–L) and summarized in Table 2.

**TABLE 2:** Quantitative lesion scoring and immunohistochemical expression of androgen receptor (AR) and heat shock protein (Hsp90) in the hepatic and renal tissues of rats in response to boldenone (BLD) and/or vitamin C (VC) treatment.

Organ	Lesion and immunoexpression		Control	Vehicle control	VC	BLD	BLD + VC
Liver	Areas	Central veins	1.43 ± 0.26	1.46 ± 0.22	1.46 ± 0.23	1.48 ± 0.24	1.51 ± 0.25
		Sinusoidal spaces	8.53 ± 0.90	8.31 ± 0.84	8.31 ± 1.12	12.75^a^ ± 1.09	11.10^a^ ^,^ ^b^ ± 0.50
		Portal blood vessels	1.77 ± 0.09	1.79 ± 0.13	1.71 ± 0.10	3.21^a^ ± 0.50	2.56^a^ ^,^ ^b^ ± 0.33
		Lipidosis	0.00 ± 0.00	0.00 ± 0.00	0.00 ± 0.00	3.44^a^ ± 1.44	2.13^a^ ^,^ ^b^ ± 0.94
		Peliosis hepatis	0.00 ± 0.00	0.00 ± 0.00	0.00 ± 0.00	5.03^a^ ± 3.31	3.09^a^ ^,^ ^b^ ± 3.08
		AR	5.66 ± 0.92	5.31 ± 0.43	5.44 ± 0.47	7.76^a^ ± 0.44	7.64^a^ ± 0.52
		Hsp90	7.52 ± 0.46	7.54 ± 0.52	7.59 ± 0.41	10.91^a^ ± 1.52	9.85^a^ ^,^ ^b^ ± 0.52
	Frequencies	Inflammatory infiltrate	0.00 ± 0.00	0.00 ± 0.00	0.00 ± 0.00	13.45^a^ ± 5.07	8.00^a^ ± 3.94
		Vacuolations	0.00 ± 0.00	0.00 ± 0.00	0.00 ± 0.00	25.58^a^ ± 5.92	18.04^a^ ^,^ ^b^ ± 2.80
		Ballooning	0.00 ± 0.00	0.00 ± 0.00	0.00 ± 0.00	9.59^a^ ± 5.09	6.88^a^ ± 4.94
		Pyknosis	0.00 ± 0.00	0.00 ± 0.00	0.00 ± 0.00	4.24^a^ ± 1.57	1.31^a^ ^,^ ^b^ ± 1.15
		Necrosis	0.00 ± 0.00	0.00 ± 0.00	0.00 ± 0.00	9.09^a^ ± 10.44	2.22^b^ ± 6.67
		Biliary hyperplasia	0.00 ± 0.00	0.00 ± 0.00	0.00 ± 0.00	5.45^a^ ± 4.63	4.44^a^ ± 5.36
		Cholestasis	0.00 ± 0.00	0.00 ± 0.00	0.00 ± 0.00	10.91^a^ ± 10.44	4.44^b^ ± 8.82
		Endothelial hypertrophy	0.00 ± 0.00	0.00 ± 0.00	0.00 ± 0.00	12.73^a^ ± 10.09	4.44^b^ ± 8.82
Kidney	Areas	AR	9.36 ± 0.44	9.52 ± 0.37	9.34 ± 0.27	13.34^a^ ± 2.70	13.04^a^ ± 2.86
		Hsp90	11.97 ± 0.75	12.25 ± 0.82	11.73 ± 0.98	19.41^a^ ± 2.71	14.22^a^ ± 1.77
	Frequencies	Glomerular congestion	0.00 ± 0.00	0.00 ± 0.00	0.00 ± 0.00	17.27^a^ ± 5.97	10.00^a^ ^,^ ^b^ ± 2.69
		Glomerular atrophy	0.00 ± 0.00	0.00 ± 0.00	0.00 ± 0.00	5.45^a^ ± 2.98	4.44^a^ ± 5.36
		Glomerular necrosis	0.00 ± 0.00	0.00 ± 0.00	0.00 ± 0.00	5.82^a^ ± 4.31	4.00^a^ ± 5.50
		Glomerular sclerosis	0.00 ± 0.00	0.00 ± 0.00	0.00 ± 0.00	1.82^a^ ± 2.60	2.22^a^ ± 3.07
		Tubular attenuation	0.00 ± 0.00	0.00 ± 0.00	0.00 ± 0.00	14.98^a^ ± 3.56	7.56^a^ ^,^ ^b^ ± 1.45
		Tubular vacuolation	0.00 ± 0.00	0.00 ± 0.00	0.00 ± 0.00	14.61^a^ ± 4.42	5.50^a^ ^,^ ^b^ ± 1.61
		Tubular pyknosis	0.00 ± 0.00	0.00 ± 0.00	0.00 ± 0.00	4.75^a^ ± 1.55	2.83^a^ ^,^ ^b^ ± 1.69
		Tubular necrosis	0.00 ± 0.00	0.00 ± 0.00	0.00 ± 0.00	2.50^a^ ± 0.84	0.68^a^ ^,^ ^b^ ± 0.66
		Cast formation	0.00 ± 0.00	0.00 ± 0.00	0.00 ± 0.00	10.01^a^ ± 4.25	3.34^a^ ^,^ ^b^ ± 2.10
		Interstitial congestion	0.00 ± 0.00	0.00 ± 0.00	0.00 ± 0.00	15.45^a^ ± 8.20	12.22^a^ ± 5.17
		Interstitial hemorrhage	0.00 ± 0.00	0.00 ± 0.00	0.00 ± 0.00	3.91^a^ ± 2.30	1.89^b^ ± 2.89
		Interstitial inflammatory infiltrate	0.00 ± 0.00	0.00 ± 0.00	0.00 ± 0.00	15.64^a^ ± 8.09	7.56^a^ ^,^ ^b^ ± 4.80

A one-way analysis of variance (ANOVA) followed by Duncan's Multiple Range test was used for statistical analysis, Values are mean ± SD for 5 samples/group.

a Significantly different compared to the control group at *p ≤ 0.05*.

b Significantly different from the BLD treated group at *p ≤ 0.05*.

### Kidneys

**FIGURE 5:**
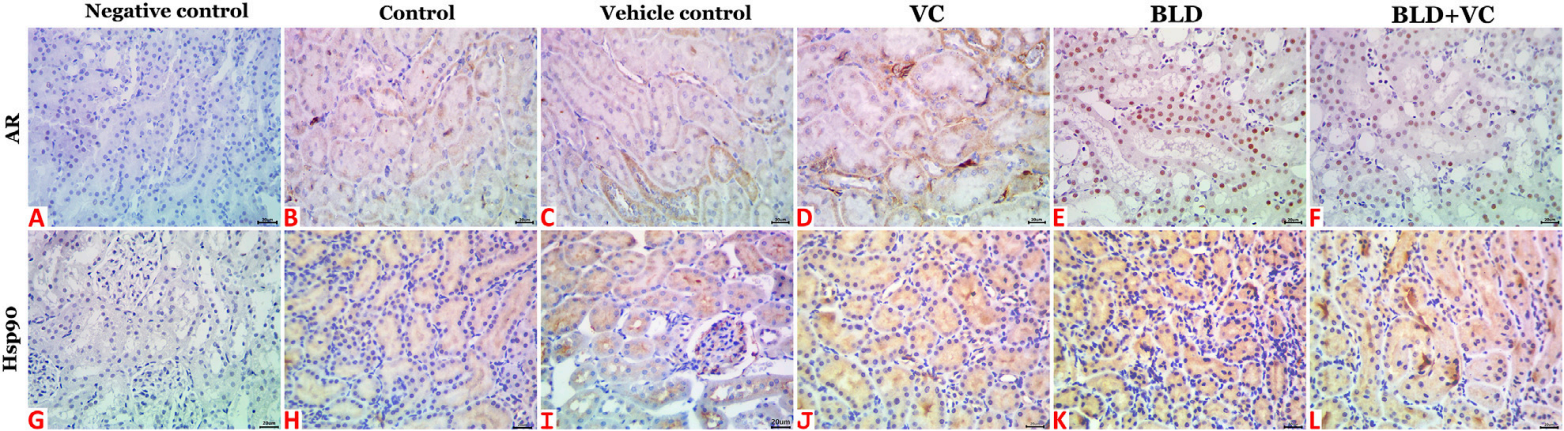
Representative photomicrograph of renal tissue sections of AR and Hsp90 immunoexpression showing a marked increase in the fractions of DAB brown areas in the BLD **(E and K)** and BLD + VC-treated **(F and L)** rats compared to the control **(B and H)**, sesame oil **(C and I)** and VC-treated **(D and J)** rats. **(A and G)** are negative controls. The scale bar is 20 microns.

The control, sesame oil, and VC-treated rats' kidneys revealed normal histological pictures (Figures 3G,H). In contrast, various nephropathic changes were seen in the BLD-injected animals. These changes involved the glomeruli (hypocellularity, atrophy, necrosis, and sclerosis), tubules (attenuation, vacuolation, pyknosis, single-cell necrosis, and debris and cast formations in their lumens), and the interstitial tissue (congestion, hemorrhage, and inflammatory infiltrates particularly with mononuclear cells) (Figures 3I,J). VC supplementation showed noticeable nephroprotective effects as the renal sections of the BLD + VC co-treated animals showed a significant reduction in the frequencies and severities of most of the BLD-induced histological changes. Yet, the kidneys did not maintain their normal histology. The most encountered lesions in this group included glomerular collapse with widening Bowman’s space, glomerulosclerosis of few glomeruli (Figure 3K), tubular vacuolations, and cast formations, interstitial congestions, and mononuclear cell infiltrate (Figure 3L). A quantitative lesion scoring in all groups was summarized in Table 2. Immunohistochemically, identical results to those of the AR and Hsp90 fractions of DAB brown areas in the hepatic tissues were obtained for all groups' renal tissues. These results were shown in Figures 5A–L and summarized in Table 2.

## Discussion

To date, scarce information is available on the possible therapeutic agents that could lessen the BLD-associated complications. Hence, the present work tested the efficacy of VC oral supplement to reduce BLD-induced hepatorenal complications using the rat model. In the current study, we used oral supplement form of VC as it is convenient to take, easily accessible, and highly effective for most people (Washio et al., 2008; DePhillipo et al., 2018). Also, despite the highly availability of VC following intraperitoneal or intravenous injection but oral supplements are still highly absorbable as previously reported (Padayatty et al., 2004; Duconge et al., 2008; Padayatty and Levine, 2016). Moreover, in the long-term treatment, instead of injection, oral administrations of solutions are more suitable to prevent tissue damages caused by multiple injections. Thus, the injection is more suitable for the cases of extreme VC deficiency and oral supplement could be more appropriate to chronic health disorders.

Activities of ALT and AST are routinely measured as diagnostic tools in assessing hepatocellular injury (Abo-EL-Sooud et al., 2018; Abd-Elhakim et al., 2019; Behairy et al., 2020a). The significant increase in ALT and AST levels in BLD-injected rats in the current study is in agreement with the earlier findings of Neamat-Allah (2014) in veal calves. Similarly, Urhausen et al. (2003) and Gabr et al. (2009) demonstrated that the ALT and AST levels considerably increased in weaned male lambs after BLD intramuscular administration. The increase in serum ALT and AST activities can be due to their release into the blood from the cytosol of the liver cells, which is confirmed by hepatic histopathological examination. Higher ALT and AST serum enzymes are a symptom of cellular leakage and cell membrane functionality within the liver (Saggu and Kumar, 2007). Oppositely, the former elevation significantly depressed in VC + BLD co-treated group. Accordingly, Oladele et al. (2020) reported that treatment with VC reduced cypermethrin-induced alterations in the biochemical activities of liver. Moreover, Bashandy and AlWasel (2011) described that VC normalized ALT and AST levels in rats' liver intoxicated with carbon tetrachloride. Besides, VC reduced necrosis and restored the normal appearance and structure of damaged hepatocytes due to emamectin benzoate exposure (Khaldoun Oularbi et al., 2017).

A significant increase in TP and ALB was recorded BLD-injected rats. Similarly, Shabir et al. (2015), revealed that TP, ALB, and globulin were significantly higher in BLD-exposed rats. Also, El-Moghazy et al. (2012) and Alm-Eldeen and Tousson (2012) verified that the TP amount was considerably increased in male rabbits after BLD injections. ASSs improve muscle size by stimulating protein development and minimizing destruction by promoting positive nitrogen balance (Guan et al., 2010). Hypoproteinemia is a known consequence of hepatic damage as the liver produces the utmost fractions of plasma-protein (Larrey, 2002). However, in the current study, the serum TP and ALB concentrations were considerably elevated after BLD injection for eight weeks. The increase in TP concentration might result from the binding of BLD to AR at the cellular level, which in turn stimulates the production of RNA and consequently increases protein formation (Orhue et al., 2005). AASs not only increase the protein synthesis in muscles but, also stimulate the production of circulating proteins (Doweiko and Nompleggi, 1991). In contrast, the co-treatment with VC significantly corrected the disturbed TP and ALB levels associated with BLD injection. Similarly, Diab et al. (2014) confirmed the hepatoprotective effect of VC against cisplatin toxicity in albino rats. This might be correlated with its considerable ability to protect hepatocytes against oxidative injury (Abdulkhaleq et al., 2018).

The current study revealed significant increases in the TC, TG, LDL-C, and VLDL-C but a substantial HDL-C level reduction in BLD-injected rats compared to the control group. Similarly, this altered lipid profile has been recorded in several case-control studies comparing those using or not using AASs or when assessing serum lipids before and after an AASs course (Kuipers et al., 1991; Hartgens et al., 2004; Li and Rabkin, 2018). Consistently, Samieinasab et al. (2015) verified the increase of the TC and TG but the HDL-C decline in female rats after ND consumption. Additionally, Tousson et al. (2018) reported a substantial increase in the cholesterol and LDL-C levels but a reduction in HDL-C levels in BLD-injected rats. It has been demonstrated that AASs' high doses increased TG levels but decreased HDL-C levels up to 70% (Achar et al., 2010), while other studies showed opposing findings (Pomara et al., 2016). This difference can be due to different sampling times, type of AASs used, and administration routes (Gårevik et al., 2012). The biochemical mechanisms by which the AASs affect HDL-C and LDL-C concentrations are not entirely understood (Li and Rabkin, 2018). Nevertheless, it is generally speculated that the induction of hepatic triglyceride lipase (HTGL) activity and modification of apolipoprotein A-I and B synthesis play essential roles in the alterations of HDL-C and LDL-C levels during the use of AASs. AASs stimulate the activity of HTGL, an enzyme that facilities catabolism of HDL (Glazer, 1991), and produces significant reductions of HDL-C and Apo-A-I concentrations (Kantor et al., 1985; Applebaum-Bowden et al., 1987). However, the mechanism behind the AASs-induced increased LDL-C levels remains to be completely elucidated. But, the overall BLD-induced hyperlipidemic condition detected here could be related to the noticeable biliary hyperplasia and cholestasis detected during histopathological examination as a strong link exists between reduced bile release and hyperlipidemic conditions (Longo et al., 2001).

The connection between VC and hypocholesterolaemia has been documented in men and guinea pigs (Kurowska et al., 2000). Herein, a significant reduction of TG, TC, LDL-C, and VLDL-C was recorded in VC + BLD co-treated rats. Similarly, Eteng et al. (2006) recorded a significant reduction in TC and VLDL-C with a non-significant rise in HDL-C in VC-treated albino Wister rats. The authors attributed VC's observed effect on serum lipids to the enzyme 7 α hydroxylase activation by VC, which improves plasma cholesterol conversion into bile acid, thereby decreasing serum cholesterol levels (Cantatore et al., 1991). The decrease of LDL-C indicates that a proper intake of VC will minimize atherosclerosis occurrence (Eteng et al., 2006).

Our results indicated that abuse of BLD as a growth promoter could contribute continuously to hepatic and renal tissues damage. These findings are consistent with Shabir et al. (2015) and Herlitz et al. (2010). Recently, Vasavan et al. (2020) observed the same alterations in urea and creatinine with ND treatment. In this respect, Loh et al. (2017) demonstrated that the elevated testosterone associated with AASs use increased the expression of aquaporins (AQPs) two four, and six in the collecting duct and AQPs-1 and sevene in the proximal convoluted tubule, consequently raised blood pressure, and increased water reabsorption resulting in hypertension and chronic kidney disease. Besides, AASs-induced direct glomerular toxicity due to increased body mass and glomerular hyperfiltration has been suggested as injury mechanisms (Parente Filho et al., 2020). The high level of urea concentration in serum is affected by high uric acid and hypophosphatemia (Gabr et al., 2009). Moreover, AASs are responsible for increasing muscle bulk and, consequently, creatinine level rises in the body (Committee on Sports Medicine and Fitness, 1997). The increase in uric acid in the current results is in harmony with some earlier studies. For instance, Neamat-Allah (2014) reported that BLD injection caused an elevation in serum creatinine level in New Zealand rabbits. Also, Salem and Alnahdi (2019) verified that the short or long-term use of the prescribed or overdose of ND altered kidney function-related biomarkers. On the contrary, Gabr et al. (2009) reported significantly reduced urea levels following BLD intramuscular injection. Increasing uric acid levels may be caused by decreased clearance due to a glomerulus filtration rate impairment or local tissue hypoxia, or an increased renal cell breakdown (Kang and Nakagawa, 2005). On the contrary, VC co-administration concurrently with BLD significantly reduced the renal function impairment. Similarly, Al-Timimi et al. (2019) signified the protective effect of VC against gentamicin-induced nephrotoxicity. This protection could be ascribed to a compensatory mechanism involving induction of antioxidant enzyme activities as a defense system by reducing ROS and increasing the nitric oxide to prevent free radical-induced cellular transformation (Moreira et al., 2014).

The significant depletion in Na^+^ and a considerable increase in K^+^ levels following BLD injection could be attributed to Na^+^/K^+^ ATPase pump inhibition, which is necessary to maintain Na^+^ and K^+^ homeostasis in eukaryotic cells. The inhibition of Na^+^/K^+^ATPase and its signaling pathways subsequently elevates the intracellular level of Ca^2+^ and Na^+,^ resulting in cardiac arrhythmia, as documented by Demiryürek and Demiryürek (2005) with high levels of steroids. The currently recorded alterations in electrolytes balance are similar to the previously reported studies by Elmasry et al. (2018) and Tousson et al. (2018). On the other hand, VC corrected the BLD-induced impaired electrolytes balance. Similarly, some earlier reports confirmed the beneficial role of VC in restoring electrolyte imbalance (Owu et al., 2016; Suleiman, 2019).

In the present study, BLD administration adversely affected antioxidant defense systems in the liver and kidney as indicated by elevated serum MDA level but decreased GSH, GPx, GST, and GSR enzyme activities. These results are compatible with the studies of Neamat-Allah (2014), El-Moghazy et al. (2012), and Tousson et al. (2018). The decline in antioxidant enzymes activities can be explained either by introducing free radicals into inactive metabolites or by the direct inhibitory influence of BLD on enzymes function (Barakat et al., 2015). The dose-dependent oxidative kidney stress and damage were recorded following the prolonged ND administration in the mice (Riezzo et al., 2014). In the earlier study, ND-treated mice showed a noticeable increase in peroxidation of lipids and reduced antioxidant enzymes, such as GPx and GSR. Prolonged administration of AASs increased ROS which damage the cells by modifying their initial chemical conformation to the double or triple bands of polyunsaturated cell membrane fatty acids (Molano et al., 1999).

Humans cannot synthesize VC because they lack one of the genes needed for its synthesis, gene encoding hepatic L-gluconolactone oxidase (Drouin et al., 2011). Thus, VC must be supplied in the human diet. Moreover, despite the ability of rats and other species like goat and reptile to normally synthesize VC in their liver (Horio et al., 1985) but Đurašević et al. (2008) confirmed that the additional intake of VC improves the antioxidative defense in rats in a dose-dependent manner. The explanation for this could be related to the mechanism of tissue accumulation of ascorbate and the balance of its alimentary and endogenous availability. Hence, the rat model has been used in various earlier studies to test the beneficial role of VC oral dosing to rescue oxidative stress conditions resulting from exposure to various toxicants (Mekkawy et al., 2020; Kader et al., 2021; Shotop and Al-Suwiti, 2021). Herein, the co-treatment with VC significantly improved the oxidative status induced by BLD injection. Such an enhancement could be attributed to VC antioxidant properties. The relieving effect of VC on oxidative status was earlier reported by Abdulkhaleq et al. (2018), Nisar et al. (2013), and El-Gendy et al. (2009). VC is a good scavenger for aqueous radicals that destroy the membrane lipids. It has a protective role in most of the cytotoxicity (Waiz et al., 2015). The mechanism by which VC reduces BLD-induced hepatic dysfunction is based on the fact that VC can reduce oxidative damage by reducing lipid peroxidation and modifying the antioxidant protection system or denoting free radical electrons and calm down their reactivity.

The current study showed a substantial rise in hepatic and renal AR receptor expressions immune-histochemically post BLD treatment, and this increase was ameliorated by VC treatment. The activation of the ARs in liver cells may increase ROS leading to hepatic cell degeneration which eventually leads to clinical signs of hepatotoxicity (Solimini et al., 2017). The impact of BLD on AR expressions in rat testes was previously reported by our earlier study (Behairy et al., 2020b). The current findings are similar to Patanè et al. (2020), who hypothesized that oxidative stress in hepatic cells had been associated with hepatotoxicity caused by AASs. Elliot et al. (2007) also demonstrated that androgens could boost AR expression in glomerular, mesangial cells, and the profibrotic cytokine at the mRNA level, thus promoting focal segmental glomerulosclerosis. Hoseini et al. (2009) also proposed that ND exposure encourages hypertrophy in proximal and distal mice tubules. Testosterone activity and ND direct action on AR may play a role in the genesis of renal fibrosis following long-term ND exposure (Brasil et al., 2015).

The current study revealed a substantial increase in Hsp90 in both hepatic and renal tissues in BLD treated group. Consistently, in trained mice injected with high doses of ND, Riezzo et al. (2014) established the Hsp90 overexpression on mesangial cells. Moreover, in the same earlier study, the authors confirmed the correlation between the dose-dependent increase in oxidative stress and overexpression of Hsp90. This was evident here by the apparent depletion of the antioxidant enzymes in the liver and kidney tissues of the BLD-injected rats. Recently, Karabulut et al. (2020) demonstrated that Hsp70 and Hsp90 expressions were increased concomitantly with increased oxidative stress in thioacetamide-treated rats. Moreover, Hsp90 expression in acetaminophen-treated mice was higher in the serum and liver samples (Wu et al., 2017). The elevated levels of these heat shock proteins could refold denatured proteins resulted from ROS-induced-oxidative stress (PeRIšIć et al., 2007). Of note, Farag et al. (2015) reported increased oxidative stress indicators in the liver BLD-injected rabbits. The increase of Hsp90 has been alleviated by VC co-treatment in BLD-injected rats. The beneficial role of VC in reducing the consequences of stress like Hsp90 overexpression has been earlier observed (McKee and Harrison, 1995; Moretti et al., 2012).

## Conclusion

The study showed that VC co-treatment significantly decreased hepatorenal impairments resulted from BLD injection for 8 weeks. Our analysis verified that VC oral dosing restored the liver and kidneys function studied parameters to normal values in BLD-injected rats. The hepatoprotective and renoprotective effects of VC can be highly associated with its antioxidant activity. Further studies of the hepatorenoprotective impacts of the derivatives of VC and other vitamins against BLD damage are warranted.

## Data Availability

The original contributions presented in the study are included in the article/Supplementary Material, further inquiries can be directed to the corresponding author.
